# Establishment of an immunogenic cell death-related model for prognostic prediction and identification of therapeutic targets in endometrial carcinoma

**DOI:** 10.18632/aging.205647

**Published:** 2024-03-08

**Authors:** Zhenran Liu, Yue Huang, Pin Zhang, Chen Yang, Yujie Wang, Yaru Yu, Huifen Xiang

**Affiliations:** 1 Department of Obstetrics and Gynecology, The First Affiliated Hospital of Anhui Medical University, Hefei 230022, Anhui, China; 2 NHC Key Laboratory of Study on Abnormal Gametes and Reproductive Tract (Anhui Medical University), Hefei 230032, Anhui, China; 3 Key Laboratory of Population Health Across Life Cycle (Anhui Medical University), Ministry of Education of the People’s Republic of China, Hefei 230032, Anhui, China

**Keywords:** endometrial carcinoma, immunogenic cell death, prediction, immune microenvironment, immunotherapy

## Abstract

Objective: Studies have firmly established the pivotal role of the immunogenic cell death (ICD) in the development of tumors. This study seeks to develop a risk model related to ICD to predict the prognosis of patients with endometrial carcinoma (EC).

Materials and Methods: RNA-seq data of EC retrieved from TCGA database were analyzed using R software. We determined clusters based on ICD-related genes (ICDRGs) expression levels. Cox and LASSO analyses were further used to build the prediction model, and its accuracy was evaluated in the train and validation sets. Finally, *in vitro* and *in vivo* experiments were conducted to confirm the impact of the high-risk gene IFNA2 on EC.

Results: Patients were sorted into two ICD clusters, with survival analysis revealing divergent prognoses between the clusters. The Cox regression analysis identified prognostic risk genes, and the LASSO analysis constructed a model based on 9 of these genes. Notably, this model displayed excellent predictive accuracy when validated. Finally, increased IFNA2 levels led to decreased vitality, proliferation, and invasiveness *in vitro*. IFNA2 also has significant tumor inhibiting effect *in vivo*.

Conclusions: The ICD-related model can accurately predict the prognosis of patients with EC, and IFNA2 may be a potential treatment target.

## INTRODUCTION

EC is a prevalent gynecological malignancy globally [1]. While many cases are detected early with a positive prognosis, the 5-year survival rate of EC has not greatly increased, and its incidence and mortality rates are increasing annually [2]. Although many studies have focused on biological subsets of EC rather than only histological types, the clinical application of the identified biomarkers remain difficult. Further exploration the development mechanism of EC is crucial to optimize treatment options and enhance patient prognosis.

The development and advancement of tumors are reliant on their interactions with the tumor microenvironment (TME), specifically the immune constituents [3]. Immunotherapy holds great promise as a treatment option for diverse solid tumors and resistant malignancies. Recently, many studies have explored new biomarkers associated with the immune microenvironment to improve the efficacy of cancer immunotherapy [4, 5]. To thoroughly explore the potential predictive significance of immune-related indicators, a wealth of data concerning the interplay between tumor-infiltrating immune cells and patients’ molecular profiles is imperative. However, the dearth of robust evidence from clinical trials hinders a comprehensive understanding of immunotherapy’s efficacy, which currently remains confined to a select cohort of patients.

ICD refers to a unique type of programmed cell death that enables individuals with functional immunity to initiate immunological reactions against antigens associated with deceased cells [6]. In the physiological state, when cells are stimulated or encounter specific conditions, they will release damage-associated molecular patterns (DAMPs) and secrete cytokines, which bind to pattern recognition receptors on dendritic cells (DCs), leading to the initiation of ICD. The death of these cells will further trigger both innate and adaptive immune responses [3, 7]. Recent studies indicate that the efficacy of specific chemotherapeutic agents relies on their capacity to trigger ICD, which transforms deceased tumor cells into vaccines with antitumor properties [8, 9]. A combination of ICD inducers and immune checkpoint inhibitors can effectively enhance the antitumor effects, by improving tumor cell immunogenicity and boosting tumor immunotherapy [10–13]. Thus, the likelihood of using ICD as a primary endpoint to identify immune biomarkers is high. A recent study of large-scale meta-analysis indicates that ICD-related immunological metagene signatures can be used to analyze the prognosis of patients with lung, breast, or ovarian malignancies [14]. Nevertheless, the association between ICD and EC requires further elucidation.

The primary aim of this research is to explore an innovative methodology, utilizing bioinformatics analysis of transcriptomic data obtained from TCGA database for patients with EC. The methodology employs ICD as a prognostic marker for evaluating the prognosis of patients with EC, while simultaneously presenting novel therapeutic targets for intervention. A novel prognostic risk model incorporating nine ICDRGs was developed to forecast the prognosis and immune microenvironment features in patients with EC. This model has the potential to facilitate informed decision-making by physicians concerning treatment options and patient outcomes in the foreseeable future.

## RESULTS

### Identification of ICDRGs and consensus clustering

**Figure 1:**
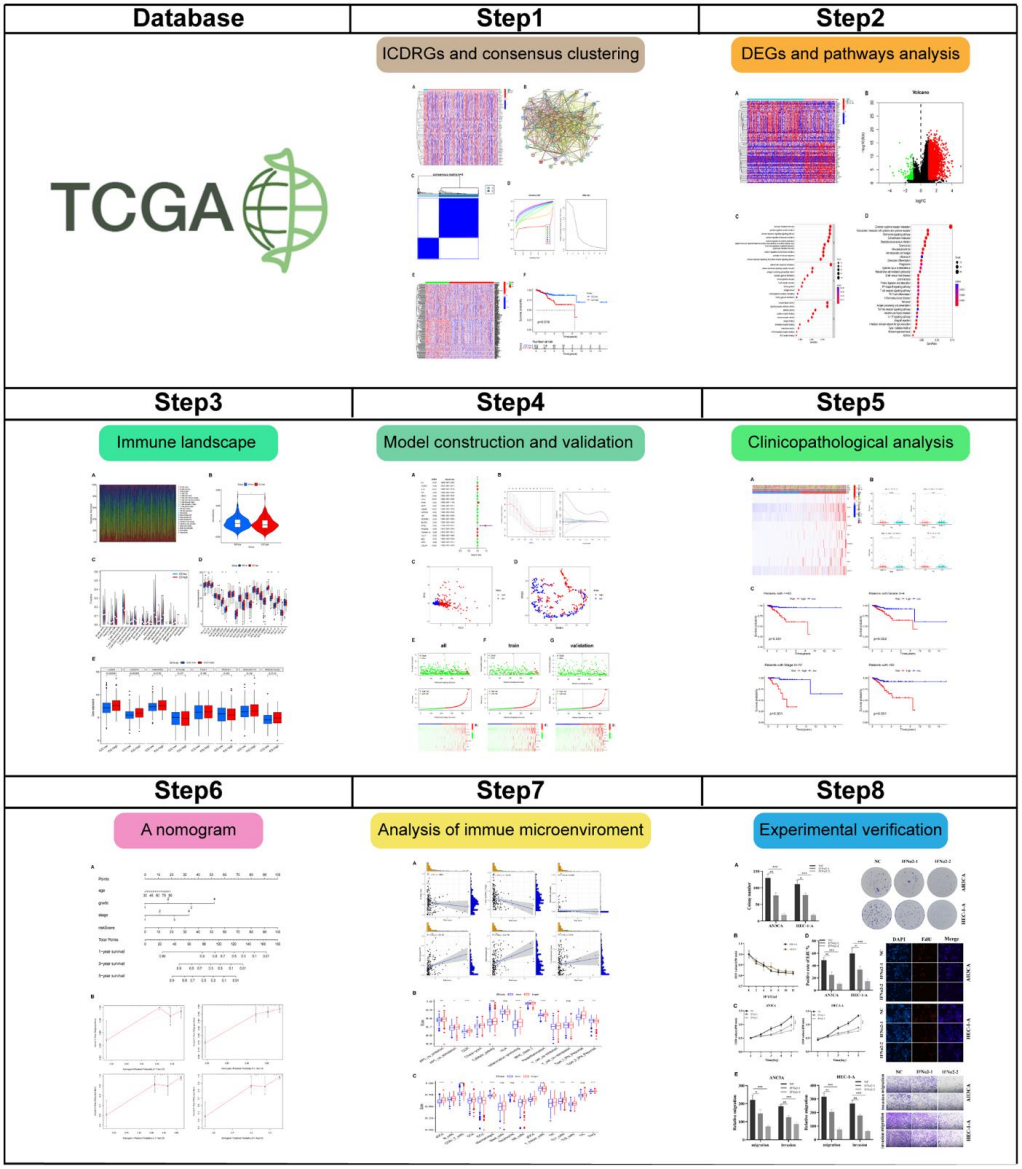
The flowchart of our study.

**Figure 2:**
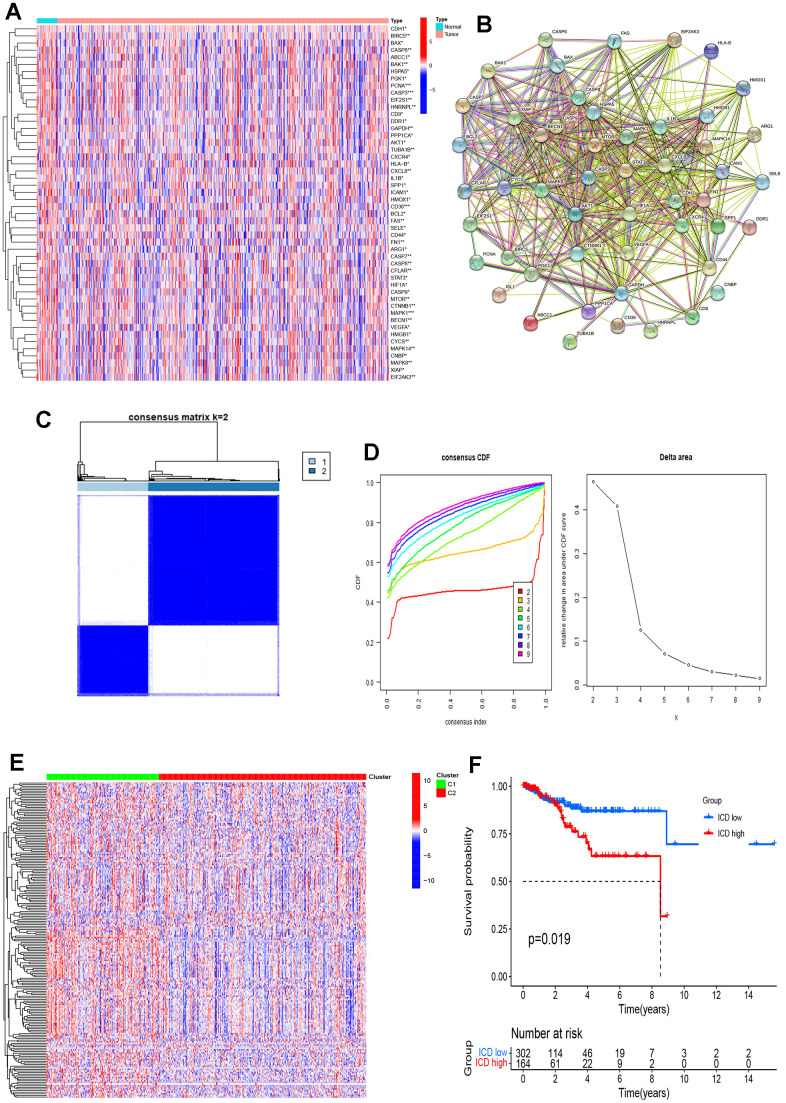
**Identification of ICDRGs and ICD subtypes by consensus clustering.** (**A**) Heatmap shows 50 ICD gene expression profiles among normal and EC samples in TCGA database; (**B**) PPI among the ICDRGs; (**C**) Heatmap depicts consensus clustering solution (k = 2) for 50 genes in 466 EC samples; (**D**) Delta area curve of consensus clustering indicates the relative change in area under the cumulative distribution function (CDF) curve for k = 2 to 10; (**E**) Heatmap of 50 ICD-related gene expressions in different groups. Red represents high expression and blue represents low expression; (**F**) KM curves of OS in ICD-high and ICD-low groups. *p< 0.05, **p< 0.01, ***p< 0.001, and ****p< 0.0001.

Figure 1 demonstrated the flow chart of this study. A dataset comprising 556 cancer samples and 33 normal samples, along with comprehensive clinical data, was obtained from The Cancer Genome Atlas (TCGA). Subsequently, 195 ICDRGs were obtained based on the GeneCards database. The results obtained revealed the top 50 significantly differentially expressed genes (DEGs), as shown in Figure 2A. An interaction network was constructed to further reveal the intricate relationships between these DEGs (Figure 2B).

We then determined the two groups associated with the ICD using consensus clustering. After k-means clustering, all cancer samples were sorted into two clusters based on various ICDRGs expression patterns (Figure 2C, 2D). Samples with different ICDRGs expression levels were clustered into different clusters (Figure 2E). Thus, two clusters represented the ICD-low and ICD-high groups, respectively. Furthermore, survival analysis demonstrated divergent clinical outcomes between the two groups. The ICD-high group displayed a poor clinical prognosis, whereas the ICD-low group exhibited a favorable clinical prognosis (Figure 2F).

### Identification of the DEGs and signal pathways between the two ICD groups

**Figure 3:**
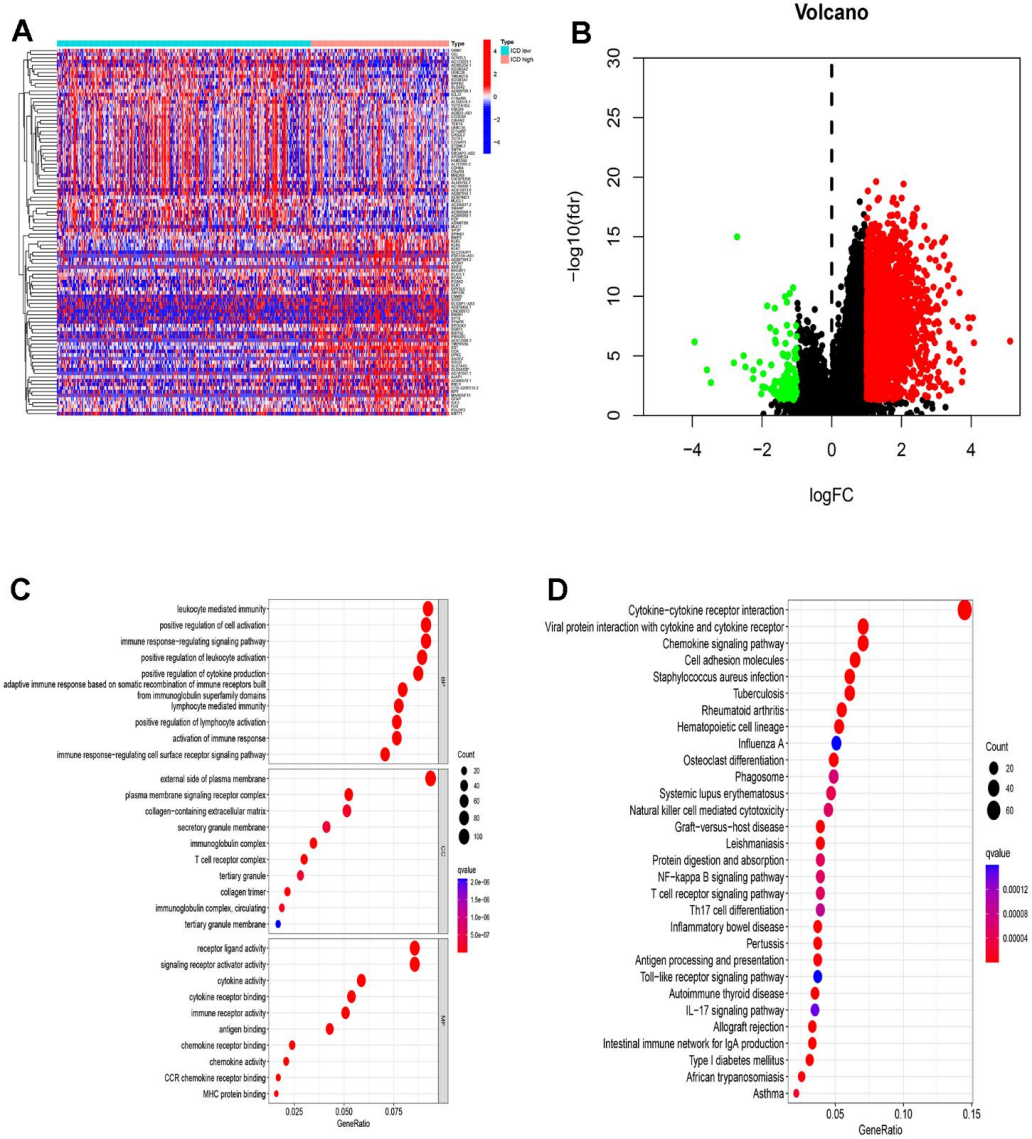
**Identification of DEGs and underlying signal pathways in different groups.** (**A**) Heatmap shows the DEGs in different groups; (**B**) Volcano plot presents the distribution of DEGs quantified between ICD-high and ICD-low groups with threshold of |log2 Fold change| > 1 and p < 0.05 in TCGA cohort; (**C**) Dots plot presents the top 10 of biological processes GO terms, cellular component GO terms, molecular function GO terms; (**D**) Dots plot presents the KEGG signaling pathway enrichment analysis. The size of the dot represents gene count, and the color of the dot represents -log10 (p. adjust-value).

To unravel the intricate molecular machinery driving divergent clinical prognoses in the two groups, we conducted a comprehensive analysis to identify novel genes and unravel the underlying signaling pathways implicated in this divergence. A total of 2,921 DEGs were detected between the two groups (Figure 3A, 3B). Furthermore, we performed Gene Ontology (GO) and Kyoto Encyclopedia of Genes and Genomes (KEGG) enrichment analyses to investigate the potential roles of these DEGs in the initiation and progression of EC. The GO enrichment analysis unveiled significant involvement of these DEGs in crucial biological processes, cellular components, and molecular functions; that is, they showed the highest upregulation in leukocyte mediated immunity, the external side of the plasma membrane, and receptor ligand activity across these three aspects (Figure 3C). Meanwhile, KEGG enrichment analysis indicated that the upregulated genes primarily enriched immune-related pathways, including cytokine-cytokine receptor interaction, chemokine signaling, viral protein interactions with cytokines and cytokine receptors and immune response-regulating signaling (Figure 3D). This finding suggests that the DEGs between different ICD groups are associated with immune-related pathways within the TME.

### TME-related immune characteristics in the two ICD groups

**Figure 4:**
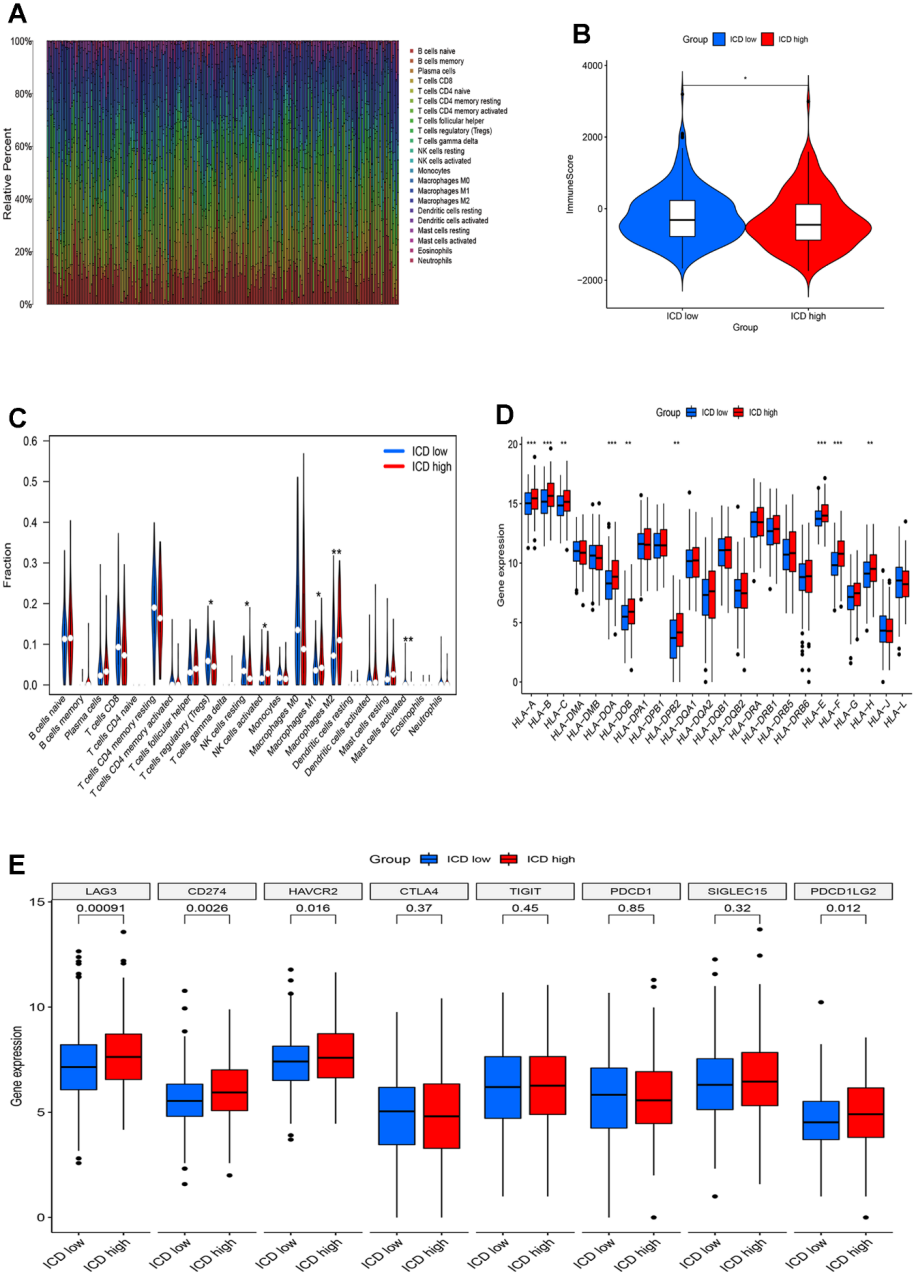
**Immune landscape of ICD-high and ICD-low groups.** (**A**) Relative proportion of immune infiltration in ICD-high and ICD-low groups; (**B**) Violin plots show the median, and quartile estimations for each immune score; (**C**) Violin plot visualizes significantly different immune cells between different groups; (**D**, **E**) Box plots present differential expression of HLA genes (**D**) and multiple immune checkpoints (**E**) between ICD-high and ICD-low groups. *p< 0.05, **p< 0.01, ***p< 0.001, and ****p< 0.0001.

Emerging evidence indicates that immune responses against tumors are influenced by ICD. We employed the CIBERS approach with the LM22 signature matrix to ascertain the infiltration status of 22 distinct immune cell types across these cancer samples (Figure 4A). Overall, analysis using the Estimation of Stromal and Immune cells in Malignant Tumor tissues using expression data (ESTIMATE) algorithm demonstrated higher immunological scores in the ICD-low group (Figure 4B). In detail, regulatory T cells (Tregs), resting and activated natural killer (NK) cells, M1 and M2 macrophages, and activated mast cells exhibited significant in infiltration levels (Figure 4C). Particularly, a notable decrease in M2 macrophage numbers was observed in the low-ICD group. Furthermore, differential expression of nine human leukocyte antigen (HLA) genes was observed between the two groups (Figure 4D). Moreover, the ICD-high group displayed elevated gene expression for four immune checkpoint genes, namely LAG3, CD274, HAVCR2, and PDCD1LG2, compared to the ICD-low group (Figure 4E). These results indicate that there are differences in immune infiltration between the two ICD groups. The ICD-low group tends to have an immune hot phenotype.

### Development and validation of an ICD-associated prognostic model

**Figure 5:**
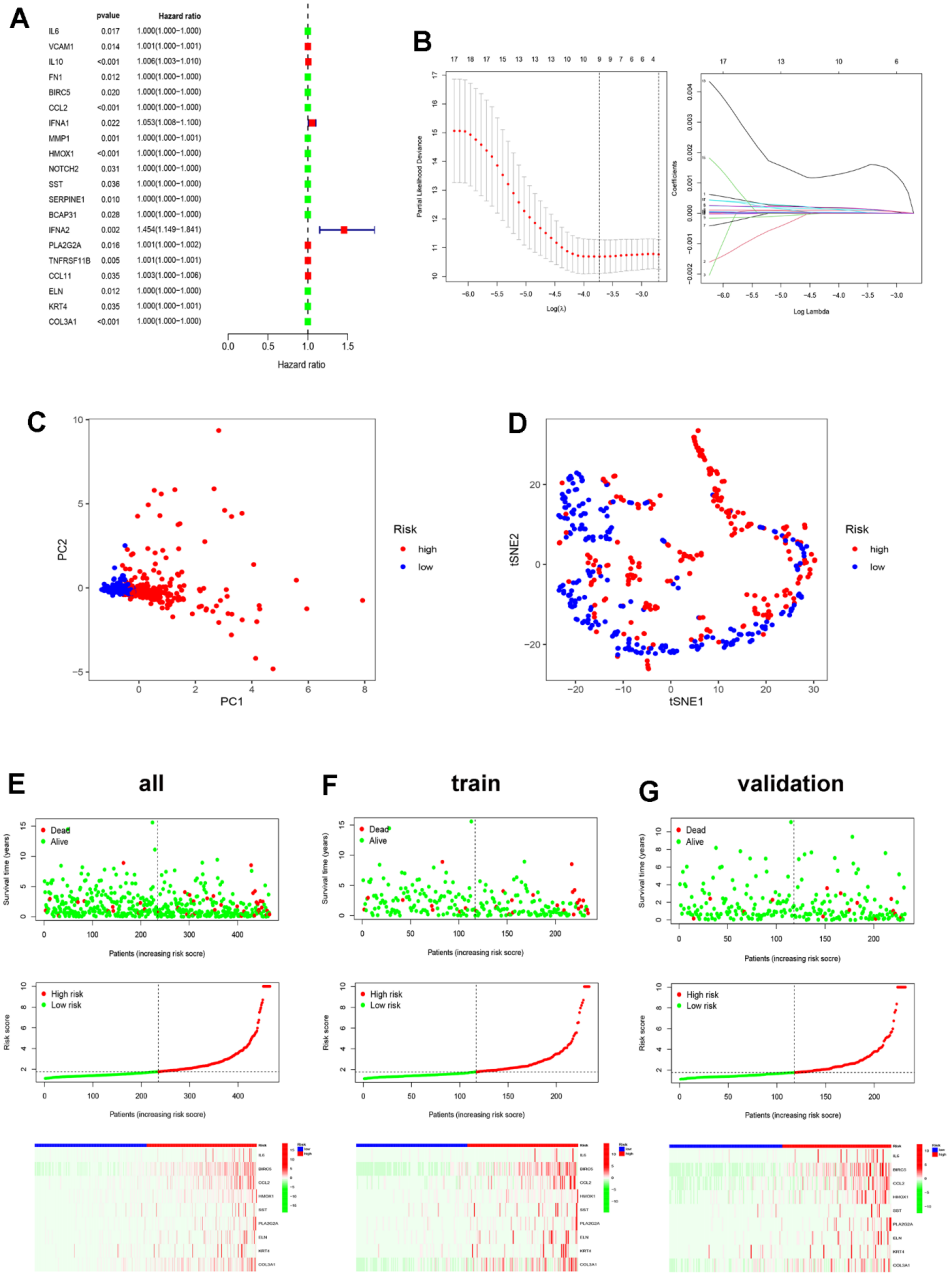
**Construction of ICD-related prognostic model.** (**A**) Univariate Cox analysis evaluates the prognostic value of the ICDRGs in terms of OS; (**B**) LASSO regression analysis to develop the prognostic model; (**C**, **D**) PCA and t-SNE analysis in the all TCGA set. The nine model genes did a more accurate job of dividing patients into two groups; (**E**–**G**) The survival status, risk score, and model gene expression in the TCGA all, train and validation sets.

Clustering with ICDRGs shows potential prognostic value. To further identify prognostic factors, of the 195 ICDRGs, 20 prognosis-related ICD genes were discerned through Univariate Cox regression (Figure 5A), namely IL6, VCAM1, IL10, FN1, BIRC5, CCL2, IFNA1, MMP1, HMOX1, NOTCH2, SST, SERPINE1, BCAP31, IFNA2, PLA2G2A, TNFRSF11B, CCL11, ELN, KRT4, and COL3A1. Subsequently, a Least Absolute Shrinkage and Selector Operation (LASSO) analysis was carried out to screen nine of these genes for constructing a risk score model (Figure 5B). The risk score formula for these nine ICDRGs is as follows:

Riskscore=0.000158532788724039*IL6+0.000123855684328424*BIRC5+0.000163885946212593*CCL2+9.06120621189331e^-5^*HMOX1+1.18478201856726e^-5^*SST+0.00141579757617881*PLA2G2A+2.45279473957131e^-5^*ELN+7.38602868946832e^-5^* KRT4+5.08402512736631e^-6^* COL3A1.

The patients were assigned a risk score using the specified formula. By leveraging the median risk score as a benchmark, patients were categorized into high- and low-risk groups. Principal component analysis (PCA) and t-distributed stochastic neighbor embedding (t-SNE) evaluations of these nine genes in the all TCGA set demonstrate the model’s effectiveness in grouping patients with EC (Figure 5C, 5D). Figure 5E displays the survival status, score trend, and gene expression level of the model’s genes in the all TCGA set. The analysis results were also performed in the train and validation sets (Figure 5F, 5G). The dot plot depicting the survival status reveals a reduced number of fatalities in the low-risk group when contrasted with the high-risk group. The heat map of gene expression levels reveals higher expression levels of the nine prognosis-related ICD genes in the high-risk group as opposed to the low-risk group.

**Figure 6:**
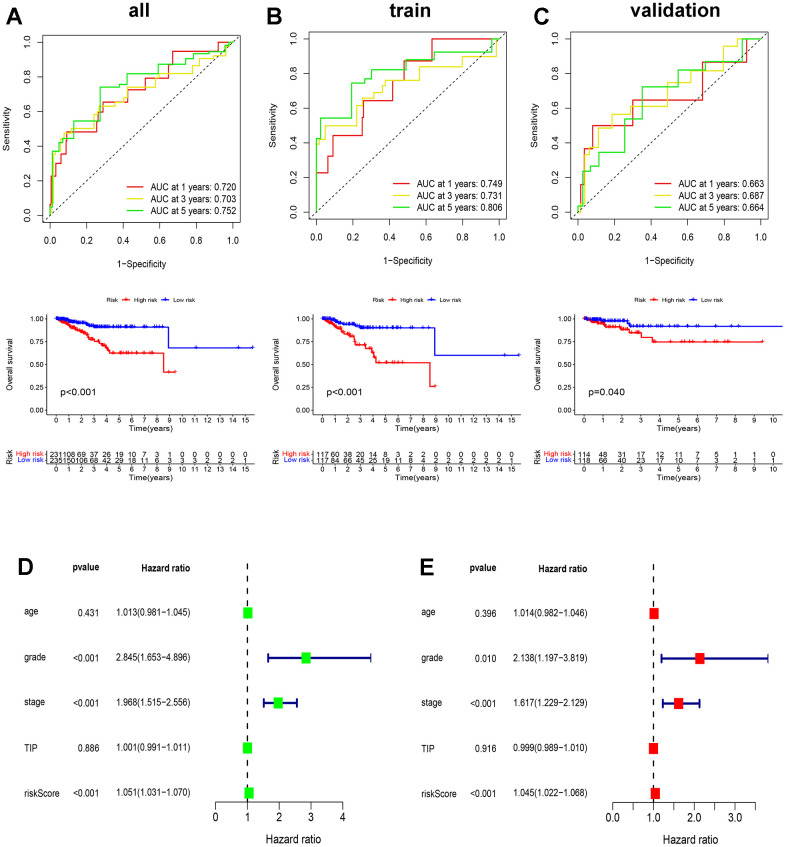
**Validation of ICD-related prognostic model.** (**A**–**C**) The ROC and OS analysis in the TCGA all (**A**), train (**B**), and validation (**C**) set; (**D**, **E**) Univariate and multivariate Cox analyses of clinical characteristics of patients with EC.

In an attempt to ascertain the predictive power of ICD in patients with EC, we conducted the receiver operating characteristic curve (ROC) and overall survival (OS) analyses on the two groups in the TCGA train, validation, and all sets. The area under the curve (AUC) values corresponding to the 1-year, 3-year, and 5-year survival rates in the TCGA all set were 0.720, 0.703, and 0.752, consecutively, and the OS value between high- and low-risk groups were significant (p<0.01) (Figure 6A), with similar results observed in the train and validation set (Figure 6B, 6C). Additionally, the ICD-related prognostic model underwent both univariate and multivariate Cox regression analyses, which aimed to uncover its potential as an independent prognostic indicator. Grade, stage, and risk score emerged as independent prognostic predictors of EC (p<0.001) (Figure 6D, 6E). These findings highlight the accuracy of the ICD-based prognostic model in predicting outcomes for both patient cohorts.

### Clinicopathological analysis of the ICD-related prognostic model in EC

**Figure 7:**
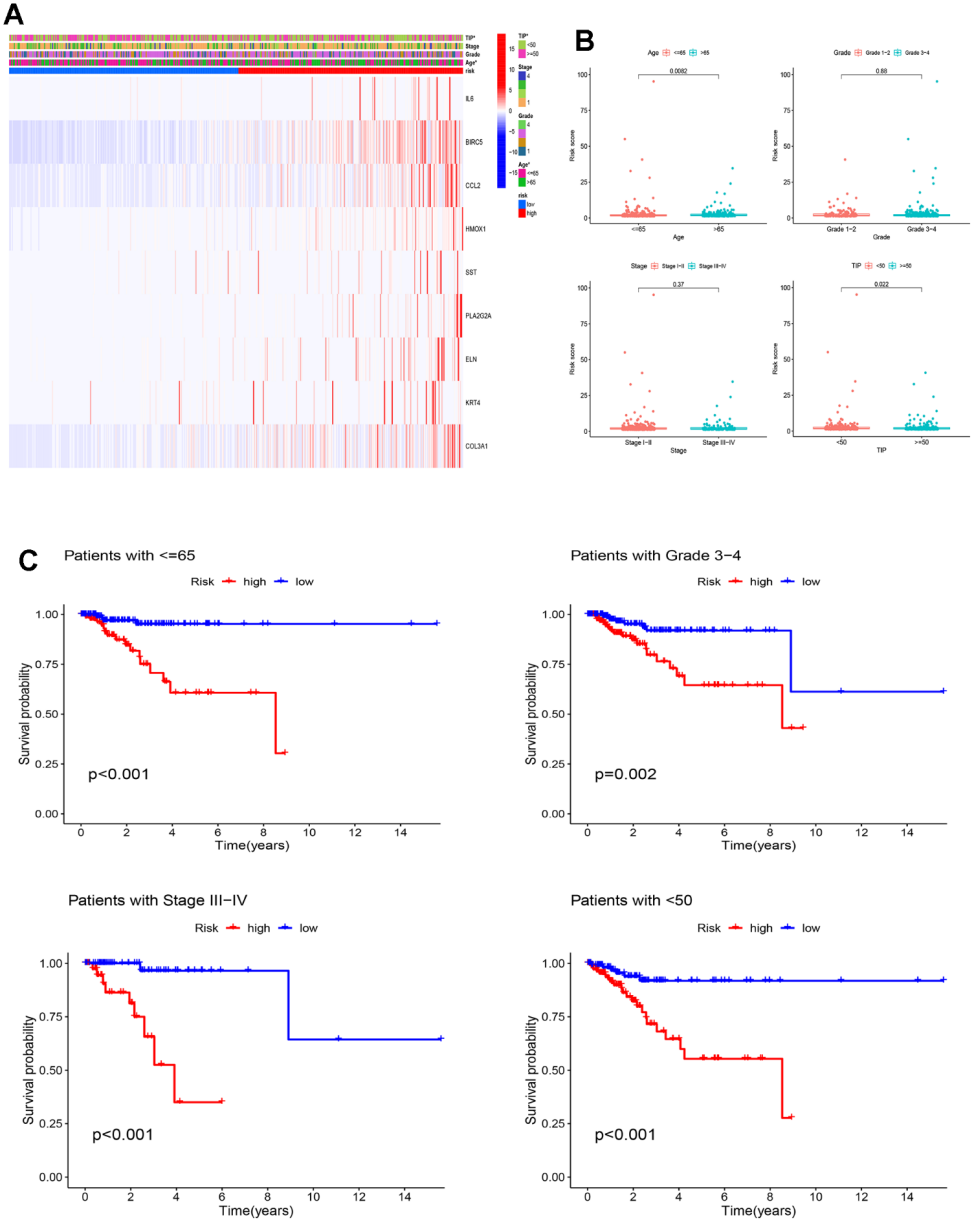
**Clinicopathological analysis of the ICD-related prognostic model.** (**A**) There were significant differences in total TIP and age between high and low-risk groups, *p < 0.05; (**B**) Relationships between the risk score and clinicopathological characteristics of EC patients; (**C**) KM curves for OS prediction in EC subtypes of Age ≤ 65 years, Grade 3-4, Stage III–IV and TIP <50.

To uncover the intricate association between the risk model we established and the diverse clinicopathological parameters, we employed a comprehensive heat map analysis. Figure 7A demonstrates profound distinctions in total TIP and age amongst the two groups (p<0.05). Furthermore, Figure 7B uncovers a striking link between the risk score and both age and TIP, while no such association is observed with grade and stage. Stratified survival analysis reveals that the high-risk group demonstrated considerably poorer OS across various EC subtypes, including subtype of patients with age≤65 (p<0.001), grade 3-4 (p=0.002), stage III-IV (p<0.001) and TIP<50 (p<0.001) (Figure 7C). These findings highlight the reliable prognostic ability of the ICD-related model predicting EC outcomes.

### A nomogram for clinical application

**Figure 8:**
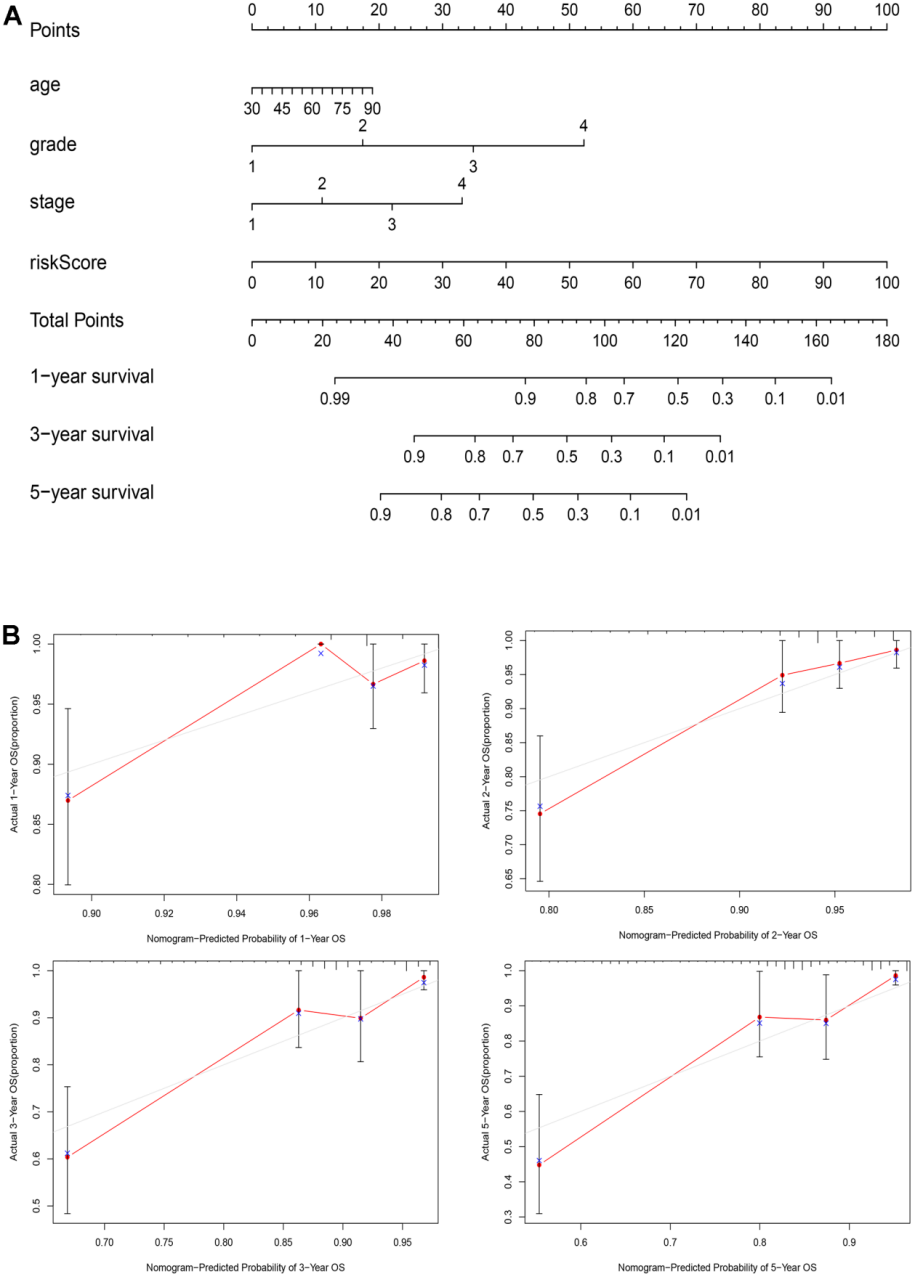
**A nomogram for clinical application.** (**A**) The nomogram based on age, grade, stage, and risk score for providing prognosis of patients with EC; (**B**) Calibration plots for the 1-year, 2-year, 3-year, and 5-year OS nomogram model.

To enhance the accuracy of prognostic assessment in patients with EC, we developed a nomogram incorporating key clinical parameters: age, grade, stage, and risk score (Figure 8A). The calibration curves of the nomogram (Figure 8B) uncovers commendable concordance between predicted and observed values across various time points, including 1-, 2-, 3-, and 5-year OS.

### Relationship between an ICD-related prognostic model and TME

**Figure 9:**
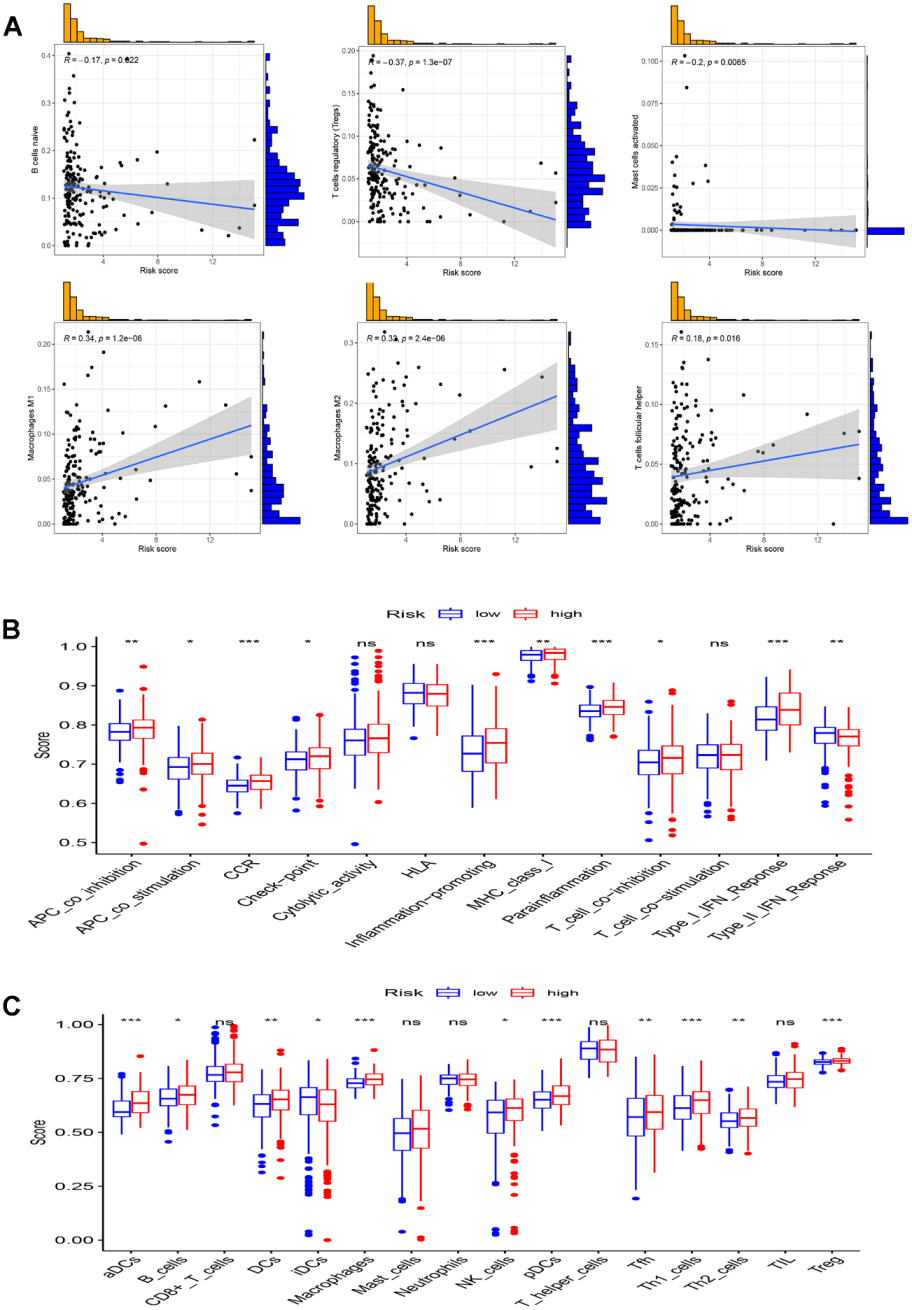
**Analysis of immune microenvironment.** (**A**) Scatter plots show the correlation of risk score with the infiltration of B cells naive, Macrophages M1, Macrophages M2, Mast cells activated, T cells follicular helper, and T cells regulatory (Tregs) in full TCGA set; (**B**, **C**) Comparison of 16 immune cell subtypes (**B**) and 13 immune function subtypes (**C**) between patients with low-risk group and high-risk group in TCGA all set. *p < 0.05, **p < 0.01, ***p < 0.001 and ns p> 0.05.

Recognizing the pivotal role of immune components in the multifaceted process of tumorigenesis and development, it is necessary to scrutinize the association of the TME with the high- and low-risk groups, as depicted in Figure 9A. The ssGSEA algorithm was employed to quantify immune infiltration levels, using scores. Notably, the high-risk group exhibited significantly higher scores for aDCs, macrophages, pDCs, Th1 cells, and Tregs in comparison to the low-risk group (Figure 9B). Furthermore, significant variations were observed in all immune function subtypes, except cytolytic activity, HLA, and T cell co-stimulation (Figure 9C).

### Gene set variation analysis (GSVA) of the two different risk groups

GSVA analysis identified a distinct pathway discrepancy between the two different risk groups. In the high-risk group, there was a significant downregulation of the KEGG pathway linked to factory transduction, while the pathways associated with cell cycle and B-cell receptor signaling exhibited significant upregulation (Supplementary Figure 1A).

### Validation of the role of IFNA2 *in vitro* and *in vivo*

**Figure 10:**
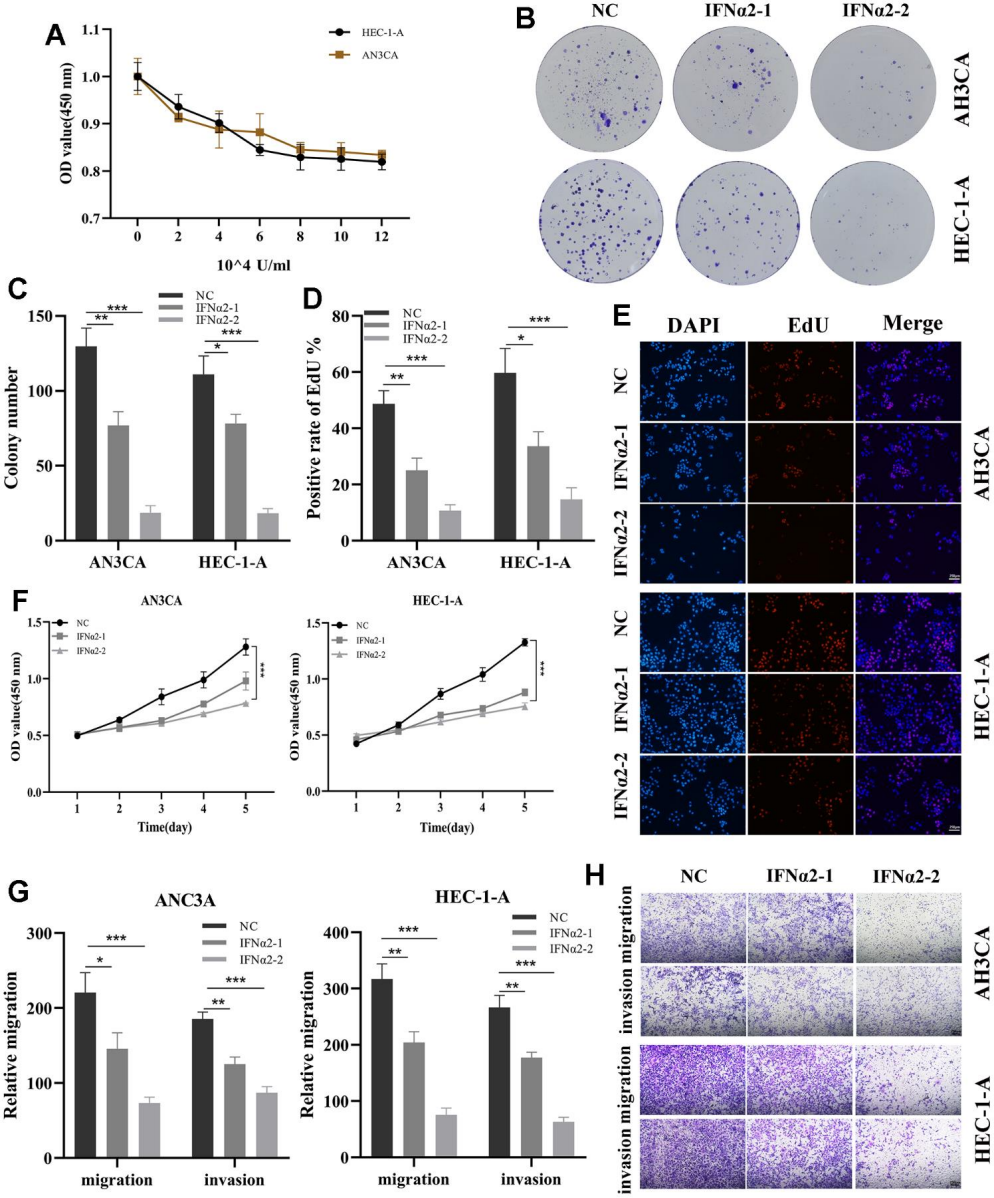
**Validation of the role of IFNA2 *in vitro* experiments.** (**A**) A CCK-8 assay was used to detect the effect of IFNα2 on the proliferation of HEC-1-A and AN3CA cells; (**B**, **C**) After treated with IFNα2 (IFNα2-1 (2×10^4^U/mL) and IFNα2-2 (8×10^4^U/mL)), the cloning ability of HEC-1-A and AN3CA cell lines decreased significantly; (**D**, **E**) EdU staining showing the effect of IFNα2 on HEC-1-A and AN3CA cells; (**F**) CCK-8 assay. After treated with IFNα2, the activity of HEC-1-A and AN3CA cell lines decreased significantly; (**G**, **H**) Transwell assay. After treated with IFNα2, the migration and invasion abilities of HEC-1-A and AN3CA cell lines were significantly decreased. *P<0.05, **P< 0.01, ***P<0.001.

IFNA2, identified as a prognostic gene through Cox regression analysis, exhibited the highest hazard ratio (HR) value, confirming its involvement in EC. In immune cells, IFNA2 facilitates the biological functions of IFNα2 in tumors by expressing and secreting it. To validate the functional role of IFNα2, *in vitro* experiments were conducted. Following a 48-hour treatment with IFNα2 at various concentration gradients in the Cell Counting Kit 8 (CCK8) experiments, a significant decrease in the proliferation capacity of both cell types is observed (Figure 10A). Therefore, we further selected two concentrations, IFNα2-1 (2×10^4^U/mL) and IFNα2-2 (8×10^4^U/mL), to treat the cells for the subsequent experiments. The results demonstrate a significant decrease in the cloning formation capacity of the cells after treatment in the cell cloning assay (Figure 10B, 10C). EdU staining revealed that the cells treated with IFNα2 had a significantly lower rate of EdU positivity compared to the control group, especially the IFNα2-2 group (Figure 10D, 10E). In addition, there was a significant decrease in the proliferative capacity of the treated cells compared to the control group as the incubation time (0h, 24h, 48h, 72h, and 96h) increased (Figure 10F). The Transwell experiment revealed a significant reduction in the migratory and invasive capabilities of both cell types treated with IFNα2 (Figure 10G, 10H). Finally, we established a BALB/c mouse subcutaneous tumor model through the injection of HEC-1-A cells. After tumor grouping, administration of IFNα2 (0 or 10000U) via intraperitoneal injection was initiated and continued until day 25. The tumor image vividly illustrates the inhibitory effects of IFNα2 on tumor growth *in vivo* (Supplementary Figure 2A–2C). These results suggest that IFNα2 has significant potential in the treatment of EC.

## DISCUSSION

EC ranks as the fourth most prevalent malignancy in women, with survival rates remaining stable over the past four decades [15]. Researchers hope to find breakthroughs in the progression of endometrial cancer, and immunotherapy has showed great promise in recent years [16]. ICD is a type of regulatory cell death that can elicit an anticancer immune response and boost the therapeutic efficacy of standard anticancer chemotherapy and radiotherapy [13, 17, 18]. Additionally, the ICD has significant prognostic value for the survival of many types of tumors, such as lung cancer, ovarian cancer, and head and neck squamous cell carcinoma [19–21]. However, the regulatory mechanism of ICD in EC and its relationship with EC prognosis remains unclear. Therefore, this study explored the expression of ICDRGs in EC, identified 20 prognostic ICDRGs, built a prognostic model based on 9 ICDRGs, and finally verified the role of the key gene IFNA2 in EC through experiments.

The focus of this study was to construct the prognostic model. In this study, we identified 50 DEGs of ICDRGs and categorized the patients into two ICD groups. Notably, the ICD-low group exhibited a favorable clinical prognosis and an activated immune microenvironment. Therefore, it is valuable to further identify the prognostic ICDRGs. Next, we identified 20 ICDRGs associated with patient outcomes by COX analysis, and constructed a risk model based on 9 ICDRGs by LASSO regression analysis. Among the 9 ICDRGs, research has demonstrated that IL6 plays a crucial role in tumor progression within the immune microenvironment [22]. BIRC5 was considered a biomarker for kidney cancer [23]. CCL2 has been reported to promote the progression of EC by regulating the recruitment of macrophages [24]. Some studies have also reported that HMOX1, SST, PLA2G2A, KRT4 and COL3A1 are associated with tumor progression and prognosis [25–29]. To validate the model’s performance, we generated ROC analysis for the train, validation and all sets. In the train and all sets, the AUC values exceeded 0.7 at 1, 3, and 5 years, with the highest value of 0.806 observed at 5 years. In the prognostic study of EC based on ferroptosis-associated genes, the research model achieved an AUC value close to 0.7 for 1-year, 3-year, and 5-year in both the training and testing cohorts [30]. This indicates that our model has certain predictive value. Additionally, our model demonstrated significant prognostic value for patients aged ≤65 years (p<0.001), patients with grade 3-4 (p=0.002), patients with stage III-IV (p<0.001), and patients with TIP<50 (p<0.001). Meanwhile, multivariable regression analysis further validated the risk score as an independent prognostic factor for EC. Despite the potential of risk scores in predicting clinical outcomes of EC, the use of a nomogram is considered a more accurate approach as it incorporates the significance of each variable. Hence, we integrated the risk score with other clinical characteristics to construct a highly accurate nomogram. These results underscore the precision of our methodology in forecasting the prognosis of EC patients.

Tumor development is strongly linked to the TME, especially immune components, which are vital for the development of effective treatments and prevention strategies [31]. Tumor-associated macrophages (TAMs) have emerged as key regulators in shaping the TME [32–34]. TAMs can adopt the M2 phenotype in EC, promoting tumor growth and invasion through various mechanisms [24, 35, 36]. Conversely, polarization of TAMs to the M1 phenotype can inhibit tumor growth [37]. Previous studies have demonstrated a negative correlation between infiltration of M2-like TAMs and the prognosis of EC [38–40]. Our findings indicate that the ICD-high group exhibited higher infiltration of M2 macrophages compared to the ICD-low group, with a positive correlation between risk score and M2 macrophages. These results align with previous research. Tumor cells evade immune system recognition through pathways such the immune checkpoint pathway [41]. Our study revealed increased expression levels of immune checkpoint genes LAG3, CD274, HAVCR2, and PDCD1LG2 the ICD-high group. High expression of LAG3 and CD274 in immune cells has been associated with poor prognosis in EC [42, 43]. The discovery of the immune system’s pivotal role in the risk model underscores its potential as a target for enhanced prognostic assessment and personalized intervention in patients with EC.

In our study, IFNA2 was identified as a prognostic gene for EC, with the highest HR value. IFNα2, expressed by IFNA2, belongs to the IFNα family and is a widely implicated immunomodulatory protein in tumor development [44]. We demonstrated the inhibitory effect of IFNA2 on tumor proliferation in both *in vivo* and *in vitro* experiments, providing further evidence for the role of IFNA2 in EC. Numerous previous studies have indicated the involvement of IFNA2 in the progression of various malignancies. Since it has been demonstrated that IFNα directly blocks cell cycle progression and triggers apoptosis, it was formerly considered to be a potential tumor therapy [45]. The effective and sustained clinical response of IFNα2 in the treatment of a variety of tumors has been expanded and confirmed [46, 47]. Recent studies have found that by altering glucose metabolism in the hepatocellular carcinoma microenvironment, IFNα can improve anti-PD-1 activity [48, 49]. Thus, our findings suggest that IFNA2 may serve as a potential factor to improve the effectiveness of immunotherapy for EC.

Our study has several limitations. Firstly, all the data analyzed in our study were obtained from the TCGA database, and further studies with larger sample sizes are needed. Due to the limitations of available data, we were unable to perform more subgroup analyses. Additionally, the lack of external datasets in the validation cohort to confirm our findings may result in biased results and a higher false-positive rate. Secondly, we only validated the potential therapeutic role of IFNA2 in EC through *in vivo* and *in vitro* experiments, hence further research is required to explore whether ICDRGs can serve as diagnostic biomarkers or therapeutic targets. Lastly, we did not investigate the underlying mechanisms of different ICDRGs in EC. Future studies should focus on elucidating the detailed mechanisms between ICD and EC.

## CONCLUSIONS

In summary, we constructed a prognosis model for EC based on ICDRGs. ICDRGs have demonstrated good reliability in prognostic prediction. We also validated the inhibitory effect of IFNA2 on EC proliferation, which could serve as a potential therapeutic target. This study enhances the prediction of prognosis in patients with EC, providing new opportunities for the diagnosis and treatment of EC.

## MATERIALS AND METHODS

### Data collection

A dataset consisting of 556 malignant and 33 normal samples was assembled by retrieving transcriptome sequencing (RNA-seq) data and comprehensive clinical information from the prestigious TCGA database (https://portal.gdc.cancer.gov). Rigorous measures were taken to ensure the integrity and reliability of the data, and patients with incomplete clinical information, such as missing survival data or outcome status, were excluded. Due to the authoritative nature of the public database, this study was exempted from requiring approval from the ethics committee.

### Identification of DEGs related to ICD

The “limma” R package was utilized to determine DEGs between malignant and normal samples, with screening a stringent requirement of |log2 (fold change)|>1 and p<0.05, facilitating the identification of molecular subtypes.

### Protein-protein interactions (PPI) network analysis

A PPI network was generated using the Search Tool for the Retrieval of Interacting Genes/Proteins (STRING) database (http://www.string-db.org/) to investigate the relationships between DEGs [50].

### ICDRGs consensus clustering

Patients with EC were stratified into two clusters based on DEGs associated with ICD using the “ConsensusClusterPlus” package in R. Two groups were identified with different ICDRGs expression levels: the ICD-high group, which had high ICDRGs expression levels, and the ICD-low group, which had low ICDRGs expression levels. Furthermore, the “survival” R package was used to generate OS curve across the two groups to assess the predictive signature of the ICDRGs.

### Construction of an ICD-associated prognostic model

A 1:1 random split was performed to create train and validation sets from the available data. The train set was employed to formulate a prognostic risk model for ICD, while the validation set was utilized to assess the credibility of this model. Initially, univariate Cox regression analysis was employed on the train set to identify prognostic genes associated with ICD. Subsequently, the “glmnet” R package’s LASSO algorithm was utilized to select candidate genes (p<0.05), with a lambda value of 1000 [51]. A multivariate Cox regression analysis was executed to validate the correlations, and the resulting coefficients were used to construct an ICD-related model for prognostic prediction. Finally, the risk score for each patient with EC was determined by applying an algorithmic equation as follows: $\text{Risk}\;\text{score}=\sum_{\text{k}=1}^{\text{n}}\text{Coef(i)}\times \text{Expr(i)}$. In this equation, Exp i denotes the degree of gene expression for ICDRGs associated with the patient prognosis with EC, while Coef i symbolizes the regression coefficient obtained through multivariate Cox regression analysis for the respective genes. Using the median risk score as a criterion, the patients with EC were categorized into groups with either high or low risk.

### Validation and evaluation of ICD-associated prognostic model

Survival analyses were carried out on not only the train set but also the validation set to evaluate the prognostic model’s precision. The prognostic model’s performance was validated by generating ROC analysis, and AUC values were derived with the assistance of the “survivalROC” R package. To compare distribution associations across different groups, we employed the R package “ggplot2” to execute PCA and t-SNE analysis. Survival analysis was employed to ascertain the predictive significance of medical indicators in measuring the difference in prognosis between the two groups. Additionally, univariate and multivariate Cox regression analyses were executed to appraise the autonomous prognostic relevance of the risk model, considering variables such as age, sex, and TNM stage.

### Performing enrichment analyses on GO terms and KEGG pathways

The “ClusterProfiler” R package was utilized to execute GO and KEGG analyses on DEGs, aiming to unveil latent signaling pathways that differentiate between various groups and employing a statistical threshold of p<0.05. Additionally, GSVA, implemented through the “GSVA” R package, was harnessed to explore the variation in biological processes and pathways among various groups.

### Estimation of the TME

Utilizing the algorithm of ESTIMATE, we appraised the composition of tumor cells from four dimensions: ESTIMATE score, immune score, stromal score, and tumor purity. Immune infiltration analysis employing the CIBERSORT algorithm compared a set of 22 immune cell types, HLA genes, and checkpoint genes.

### Developing a prognostic nomogram

To enhance the accuracy of evaluating the prognostic model’s predictive capability, we developed a nomogram that incorporates the clinical parameters of patients with EC. This nomogram was constructed using age, grading, staging, and risk score derived from multivariate Cox regression analyses.

### Cell culture

The EC cell lines HEC-1-A and AN3CA were procured from the biobank at the First Affiliated Hospital of Nanjing Medical University. HEC-1-A cells were nurtured in McCoys’ 5A medium (ProCell, Wuhan, China) fortified with 10% fetal bovine serum (FBS; VivaCell, Shanghai, China). AN3CA cells underwent cultivation in a medium consisting of DMEM (Gibco, BRL, USA) enriched with 10% FBS. All cells were maintained at 37° C with 5% CO_2_ and passaged upon reaching 80% confluence for subsequent experiments.

### CCK8 assay

Upon completing the digestion, centrifugation, and resuspension steps, the cells were meticulously dispensed into a 96-well plate at a precise density of 5×10^3^ cells per well. Subsequently, the plate underwent incubation, utilizing a 10 μL infusion of the CCK-8 labeling reagent (CT0001-B; Sparklade) for a duration of 2 hours. The cells were treated with CCK-8 (Sparklade, Shandong, China) for 0, 24, 48, 72, and 96 h. Finally, to determine the viability of the cells, we determined the optical density of the samples at the wavelength of 450 nanometers employing an enzyme immunoassay reader.

### Colony formation assay

A population of 1000 cells was distributed into each well of a six-well plate, followed by an incubation period of 2-3 weeks. Once cell clones became visible, the cells were delicately rinsed with a phosphate-buffered saline (PBS) solution, ensuring gentle removal of any residual debris. Subsequently, they were meticulously fixed with a 4% paraformaldehyde (PFA) solution for a precisely timed period of 15 minutes. Following another PBS rinse, the cells were subjected to staining using crystal violet for a duration of 20 minutes. After the staining process, the cells were left to air-dry before being counted using an appropriate method.

### EdU staining

The experiment involved culturing cells in 96-well plates with a bustling population of 5000 cells per individual well, followed by a 48-hour treatment period. Negative and treated cells were then stained with EdU solution for 2 hours. After treating the cells with a 4% PFA fixative for 15 minutes, they undergo a triple wash in PBS solution containing 3% bovine serum albumin. Afterward, permeabilization was achieved by treating the cells with PBS enriched 0.3% Triton X-100 for 15 minutes. Subsequently, the plates were incubated with freshly prepared Click reaction solution (50uL) for 30 minutes at room temperature in the absence of light exposure. EdU staining was executed using the BeyoClick EdU-594 kit (C0078S; Beyotime, Shanghai, China), while DAPI staining for nuclei was carried out using DAPI Staining Solution (C1005; Beyotime). Utilizing an inverted fluorescence microscope, the images were captured and recorded.

### Transwell assay

Transwell chambers were utilized to conduct cell migration and invasion assays. For the invasion experiment, the upper chamber was coated with Matrigel solution. The subsequent steps were identical for both assays. In the upper chamber, non-serum cultured cells were digested after 24 hours and seeded at a concentration of 2×10^4^ cells/well in 200 μL of non-serum medium. Simultaneously, the bottom chamber was filled with 600 μL of 10% serum medium. Upon fixation with a 4% PFA solution and subsequent staining with a 0.1% crystal violet solution, the cells were quantified.

### Mouse xenograft model

We obtained female BALB/c nude mice (5 weeks old) from Hangzhou Ziyuan Experimental Animal Technology (Zhejiang, China). The mice were maintained under a 12-hour light and 12-hour dark cycle, allowing them to freely access food and water throughout the dark period. 1×10^7^ HEC-1-A cells with mixed PBS and Matrigel solution 1:1 were injected into the subcutaneous area of the right wing of nude mice. The tumor volume is calculated using the formula: volume = 1/2 (Length×Width^2^). Tumors with volumes approximately 100mm^3^ were randomly divided into two groups, with five tumors in each group and treated with IFNα2 (10000U) every two days. Tumor size was measured with a caliper every 5 days. The mice were sacrificed 25 days later, the tumors were surgically removed, and the volume and weight of the tumors were measured.

### Statistical analysis

The R program (version 4.1.0) was utilized for statistical analysis in the medical study. The Wilcoxon test compared two groups, while Spearman’s or Pearson’s correlation analyzed the correlation matrices. Survival differences were assessed using Kaplan-Meier (KM) curves and the log-rank test, taking into account statistical significance at a p-value threshold of less than 0.05.

## Supplementary Material

Supplementary Figures
