# Supplementary material for: Establishment of an immunogenic cell death-related model for prognostic prediction and identification of therapeutic targets in endometrial carcinoma
Source: Aging (Albany NY). 2024 Mar 8;16(5):4920–42. doi: 10.18632/aging.205647 (PMC10968672; doi:10.18632/aging.205647)
Supplement: Supplementary Figures [file aging-16-205647-s001.pdf]

SUPPLEMENTARY FIGURES

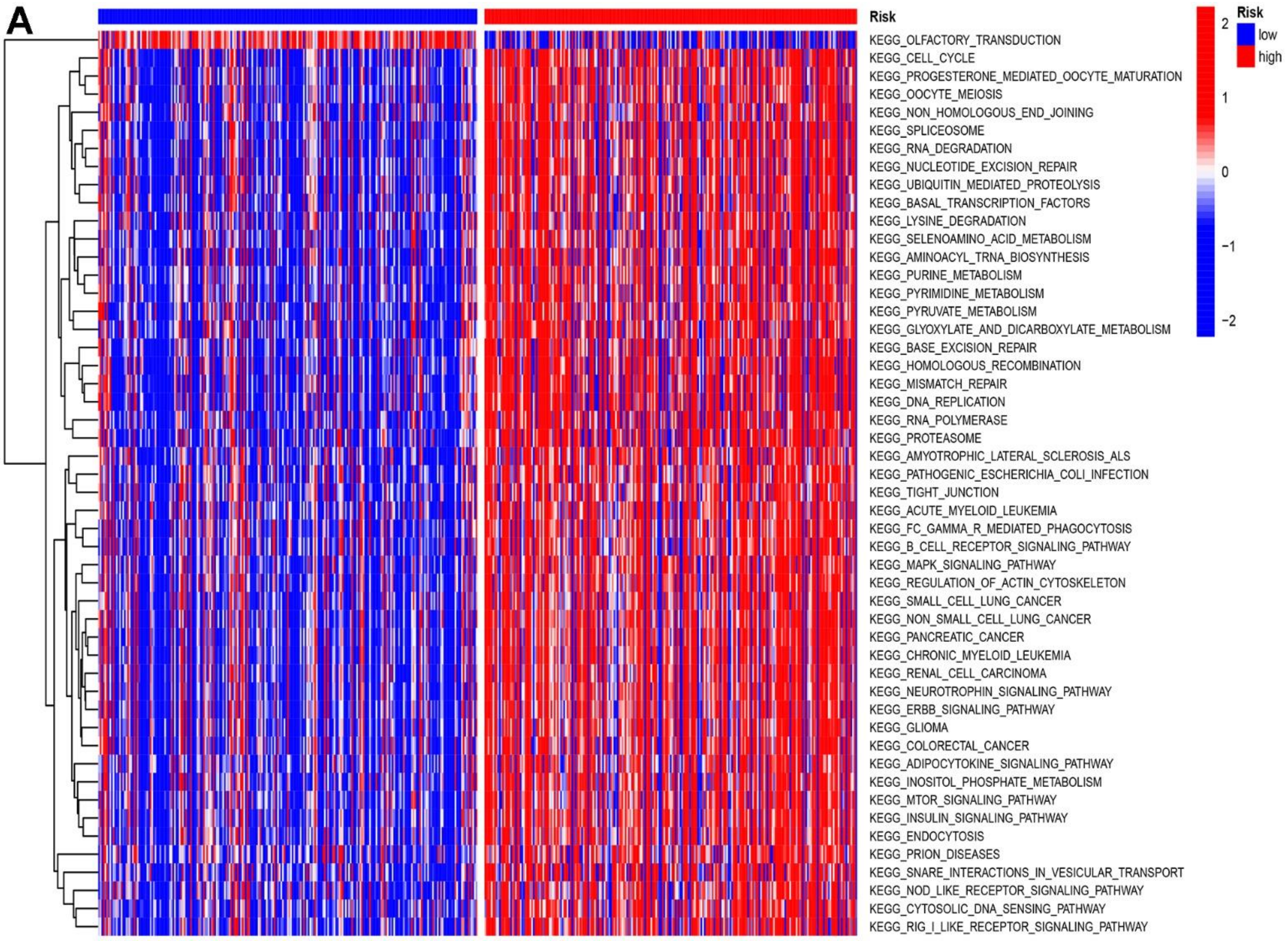

Supplementary Figure 1. GSVA analysis. (**A**) GSVA of two different risk groups based on the KEGG pathway.

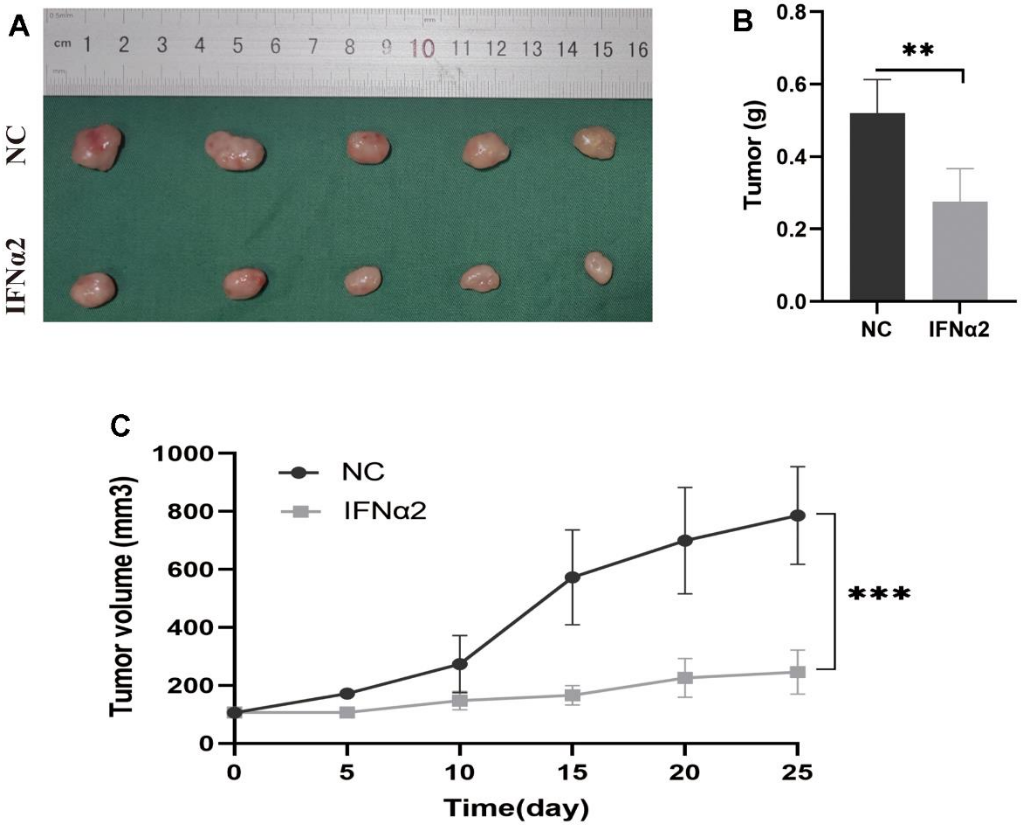

Supplementary Figure 2. IFNA2 inhibited EC cell proliferation *in vivo*. (**A**) Tumor photos of different groups of nude mice in the NC group and IFNA2 treatment (10000U/2 days) group. (**B**, **C**) Average body weight (**B**) and tumor size (**C**) were used to observe the tumors. ** $p < 0.01$ and *** $p < 0.001$.
